# Characterization of the Non-Polio Enterovirus Infections Associated with Acute Flaccid Paralysis in South-Western India

**DOI:** 10.1371/journal.pone.0061650

**Published:** 2013-04-22

**Authors:** Rongala Laxmivandana, Prasanna Yergolkar, Varanasi Gopalkrishna, Shobha D. Chitambar

**Affiliations:** 1 Enteric Viruses Group, National Institute of Virology, Pune, India; 2 National Institute of Virology, Bangalore-unit, Bangalore, India; Columbia University, United States of America

## Abstract

Non-polio enteroviruses (NPEVs) have been reported frequently in association with acute flaccid paralysis (AFP) cases during Polio Surveillance Programs (PSPs) worldwide. However, there is limited understanding on the attributes of their infections. This study reports characteristics of NPEVs isolated from AFP cases, investigated during PSPs held in 2009–2010, in Karnataka and Kerala states of south-western India having varied climatic conditions. NPEV cell culture isolates derived from stool specimens that were collected from 422 of 2186 AFP cases (<1–14 years age) and 17 of 41 asymptomatic contacts; and details of all AFP cases/contacts were obtained from National Polio Laboratory, Bangalore. The distribution of NPEV infections among AFP cases and circulation pattern of NPEV strains were determined by statistical analysis of the data. Genotyping of all NPEV isolates was carried out by partial VP1 gene sequencing and phylogenetic analysis. NPEV positive AFP cases were significantly higher in children aged <2 years; with residual paralysis; in summer months; and in regions with relatively hot climate. Genotyping of NPEVs identified predominance of human enteroviruses (HEV)-B species [81.9%—Echoviruses (E): 57.3%; coxsackieviruses (CV) B: 15%; numbered EVs: 8.9%; CVA9: 0.7%] and low levels of HEV-A [14.5%—CVA: 6%; numbered EVs: 8.5%] and HEV-C [3.6%—CVA: 2.6%; numbered EVs: 1%] species, encompassing 63 genotypes. EV76 (6.3%) and each of E3, CVB3 and E9 (4.97%) were found frequently during 2009 while E11 (6.7%), CVB1 (6.1%), E7 (5.1%) and E20 (5.1%) were detected commonly in 2010. A marked proportion of AFP cases from children aged <2 years; presenting with fever; and from north and south interior parts of Karnataka state was detected with E/numbered EVs than that found with CVA/CVB. This study highlights the extensive genetic diversity and diverse circulation patterns of NPEV strains in AFP cases from different populations and climatic conditions.

## Introduction

Acute flaccid paralysis (AFP) is a clinical syndrome known to be manifested in humans by infectious (bacterial or viral) or non-infectious (metabolic disorders or trauma or metal toxicity) causes or post-infectious autoimmune condition (eg: Guillian Barre syndrome [GBS]) [1], [2]. Among bacterial agents—*Borrelia burgdorferi*, *Corynebacterium diphtheriae, Clostridium botulinum* and *Mycoplasma pneumoniae* and viral agents—enterovirus (EV), flavivirus, herpes virus, rabies virus and tick borne encephalitis virus have been found frequently [3], [4].

EVs, the members of the genus *Enterovirus*, family *Picornaviridae* are non-enveloped icosahedral particles, 27–30 nm in diameter. The genome consists of a positive sense single stranded RNA molecule, 7400–7500 nucleotides in length that encodes a large poly-protein. The post-translational cleavage products include four structural (VP1, VP2, VP3 and VP4) and seven non-structural (2A, 2B, 2C, 3A, 3B, 3C and 3D) viral proteins [5].

EVs spread mainly by the faecal-oral route and rarely by respiratory route [5]. Based on the pathogenicity in humans and experimental animals, EVs were classified into four groups, polioviruses (PVs), coxsackieviruses A (CVA), coxsackieviruses B (CVB), and echoviruses (E) [6]. Recently, human enteroviruses (HEVs) have been subdivided into four species (HEV-A, HEV-B, HEV-C and HEV-D) [7] by phylogenetic analysis of variable region of the genome, among which more than 100 genotypes have been described.

EVs cause a large number of asymptomatic infections and are the leading etiological agents of aseptic meningitis. They also cause serious diseases of nervous system like AFP and encephalitis [5], [8], [9]. Among EVs, PVs are known to be the main cause of AFP. Though PV has been eradicated in many countries due to intensive oral polio vaccination (OPV) program, several non-polio AFP cases are being reported annually, worldwide [10], [11]. Isolation of non-polio enteroviruses (NPEVs) is documented frequently from such cases [12] as a part of the requirement of Polio Surveillance Programs (PSPs), during the process of PV detection. However, they have no programmatic significance and are not studied further. In India, characterization of NPEVs from AFP cases has been reported rarely [13], [14], [15], although their occurrence has been recorded often [16]. Moreover, there is limited understanding on the characteristics of NPEV infections in the backdrop of meteorological zones. The present study was conducted to examine different features associated with NPEV infections among AFP cases investigated in PSPs held during 2009–2010 in different meteorological zones of Karnataka and Kerala states in south-western India. Further, molecular characterization of the NPEV isolates was carried out to identify the genotypes, their diversity and circulation pattern. In addition to this, NPEV strains isolated from asymptomatic contacts of AFP cases from the same region, during the same period were also analyzed.

## Materials and Methods

### Ethics statement

As this study involved use of cell culture isolates of viruses recovered from stool specimens of AFP cases/asymptomatic contacts, investigated at the National Polio Laboratory (NPL), National Institute of Virology (NIV), Bangalore-unit, the requirement of informed consent was waived off by the Institutional Ethical Committee as per the Indian Council of Medical Research (ICMR) Guidelines 2006 [17].

### Case selection criteria and specimens

A total of 2186 AFP cases comprising 1228 males and 958 females, investigated in 2009 (n = 1048) and 2010 (n = 1138), in Karnataka (n = 1530) and Kerala (n = 656) states of south-western India under the National Polio Surveillance Project (NPSP) at NPL, NIV Bangalore-unit, were included in the study. All cases were ≤14 years of age, recipients of ≥3 doses of OPV, and found to present with clinical features of AFP as defined in the program [16]. Virus isolation was carried out in human rhabdosarcoma (RD) and human poliovirus receptor-CD155 expressing recombinant murine (L20B) cell lines from chloroform extracted stool specimens according to the World Health Organization (WHO) protocols accepted for the program [18]. The stool specimens producing *cytopathic effect* (CPE) only in RD cells and not in L20B cells were considered to contain NPEVs [19]. The specimens producing CPE in L20B cell line and also containing mixtures of polio vaccine viruses and NPEVs (usually CVA viruses [20]) were excluded from the study. Of the 2186 stool specimens processed, a total of 422 NPEVs were isolated in RD cell line, 350 from Karnataka (n = 194 in 2009, n = 156 in 2010) and 72 from Kerala (n = 33 in 2009, n = 39 in 2010) states. Seventeen of 41 stool specimens collected from asymptomatic contacts of AFP cases from Karnataka (n = 14 in 2009, n = 3 in 2010) state also yielded NPEVs. All NPEV isolates were stored at −20°C, and shipped on wet ice to NIV, Pune for further processing. The details of AFP cases were obtained from the case line lists processed at NPL, NIV Bangalore.

### RNA extraction and RT-PCR for genotyping

Viral RNA was extracted from cell culture supernatants by using Magmax RNA isolation kit (Ambion Inc, USA), according to the manufacturer's instructions. Extracted single stranded RNA (5 µl) was then reverse transcribed to complementary DNA (cDNA) using 0.5 µl of 50 U/µl Moloney Murine Leukemia Virus (M-MuLV) reverse transcriptase (RT) enzyme (Roche, USA) and a reaction mix containing 1.5 µl of 5× RT reaction buffer, 1 µl of 100 mM Dithioerythritol (DTT), 0.5 µl of 50 µM VP1 gene specific 222 reverse primer (nucleotide position: 2969–2951) [21], 1 µl of 10 mM dNTPs and 0.5 µl of 40 U/µl RNase inhibitor in a total volume of 10 µl at 43°C for 60 minutes (min). Ten microlitres of cDNA was added to 40 µl of PCR mix containing 0.5 µl of 3 U/µl of Chromous Hifi polymerase (Chromous Biotech Pvt. Ltd), 5 µl of 10× Chromous Hifi PCR buffer, 1 µl of 10 mM dNTPs, 31.5 µl nuclease free H_2_O and 1 µl of 50 µM each of 224 forward (nucleotide position: 1977–1996) and 222 reverse (nucleotide position: 2969–2951) degenerate primers targeting the nucleotide region: 1977–2969 [21] for generation of a product of 992 bp. PCR was carried out with an initial denaturation at 95°C for 2 min followed by 35 cycles of amplification (95°C for 2 seconds (s), 50°C for 10 s, and 72°C for 2 s) and a final extension at 72°C for 5 min. Three microlitres of the first PCR product was added to 47 µl of reaction mix containing the PCR reagents, 38.5 µl nuclease free H_2_O and 1 µl of 40 µM each of AN89 forward (nucleotide position: 2602–2627) and AN88 reverse (nucleotide position: 2977–2951) primers targeting the proximal part of VP1 region (nucleotide position: 2602–2977) [21] for generation of a product of 375 bp in the second round of amplification. PCR was carried out with an initial denaturation at 95°C for 2 min followed by 35 cycles of amplification (95°C for 2 s, 55°C for 10 s, and 72°C for 2 s) and a final extension at 72°C for 5 min. All of the final PCR products were analyzed on ethidium bromide stained agarose gel (2%), visualized under UV light, excised from gel and purified using the QIAquick gel extraction kit (Qiagen, Hilden co., Germany).

### Nucleotide sequencing and phylogenetic analysis

All amplified products were sequenced using ABI PRISM Big Dye Terminator cycle sequencing ready reaction kit (Applied Biosystems, USA) on an automated DNA sequencer (ABI PRISM 3100 Genetic analyser, Applied Biosystems, USA). All of the partial VP1 sequences obtained were used as query sequences for comparison with those available in the GenBank and each product (isolate) was assigned the genotype that gave the highest identity score, according to the sequence similarity criteria defined earlier [22]. Nucleotide sequences of partial VP1 gene were aligned with the sequences of reference strains available in GenBank by using CLUSTAL-W program. Phylogenetic analysis was carried out in MEGA version 5.05 software package by using p-distance and neighbor-joining algorithm [23]. The reliability of the phylogenetic trees was confirmed by using the bootstrap test (1000 replications).

### Statistical Analysis

Statistical analysis was carried out using the Open Epi 2.3.1 version [24]. The proportions were compared using chi-squared test/mid-P exact test. The p-values<0.05 were considered statistically significant.

### Nucleotide sequence accession numbers

The VP1 sequences of the representative strains of all 64 genotypes identified in the present study were deposited in the GenBank database under the accession numbers JX476125–JX476271 and JX519427–JX519428.

## Results

### Gender and age dependent distribution of NPEV positivity among AFP cases

**Figure 1:**
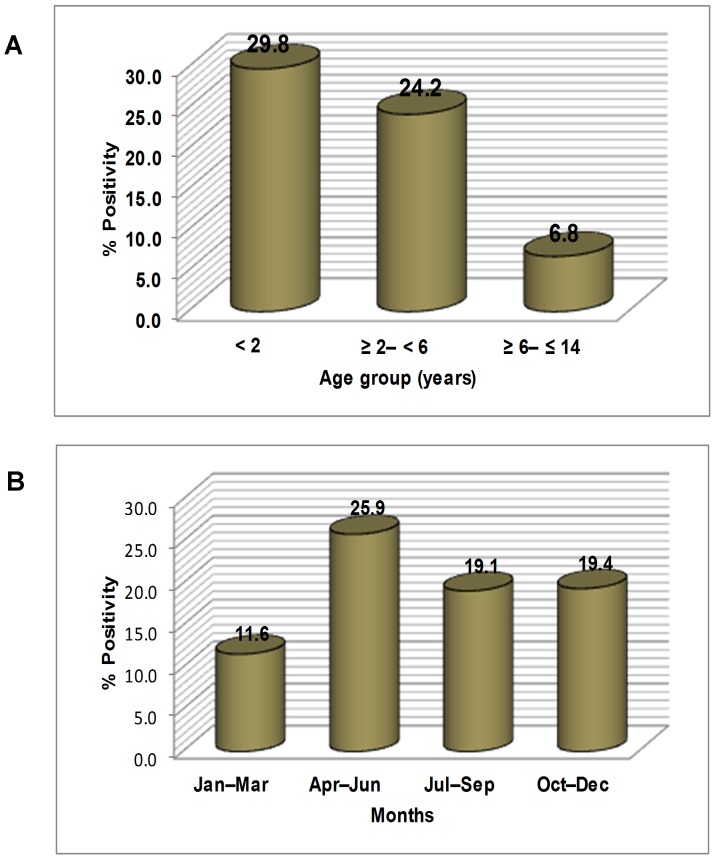
NPEV positivity in AFP cases of the present study. (A) Age-wise distribution. (B) Month-wise distribution.

NPEV infections were detected in both males and females (228/1228; 18.6% vs 194/958; 20.3%, p>0.05) and were found to be significantly higher in children aged <2 years (151/506; 29.8%) than in pre-school children aged ≥2–<6 years (218/899; 24.2%, p<0.03) and children aged ≥6–≤14 years (53/781; 6.8%, p<0.0000001). The latter group showed significantly lower NPEV positivity than pre-school children (p<0.0000001) (Figure 1A). Among children aged <2 years, the age group of ≥1–<2 years showed higher NPEV positivity (117/371; 31.5%) than the age group of <1 year (34/135; 25.2%).

### Analysis of clinical features of NPEV infected AFP cases

Analysis of available clinical data showed that NPEV positivity was not different in cases with or without fever at the onset of paralysis (178/934; 19% vs 190/1063; 17.9%). Similar results were obtained for the cases with or without asymmetric paralysis (174/934; 18.7% vs 170/930; 18.3%) (p>0.05). In a total of 312 AFP cases that were followed after 60 days of onset of paralysis, NPEV positivity was significantly high in AFP cases with residual paralysis as compared to recovered cases (21/124; 16.9% vs 17/188; 9%, p<0.04).

### Seasonality and climatologic factors associated with NPEV positivity in AFP cases

Though NPEV positive AFP cases were detected throughout the year, significantly more number of cases were found during April–June (summer months) (146/564; 25.9%) than in July–September (monsoon months) (122/638; 19.1%, p<0.005), October–December (post-monsoon months) (99/510; 19.4%, p<0.02) and January–March (winter months) (55/474; 12%, p<0.0000001). Further, the winter months showed significantly lower NPEV positivity than monsoon/post-monsoon months (p<0.0008) (Figure 1B).

**Figure 2:**
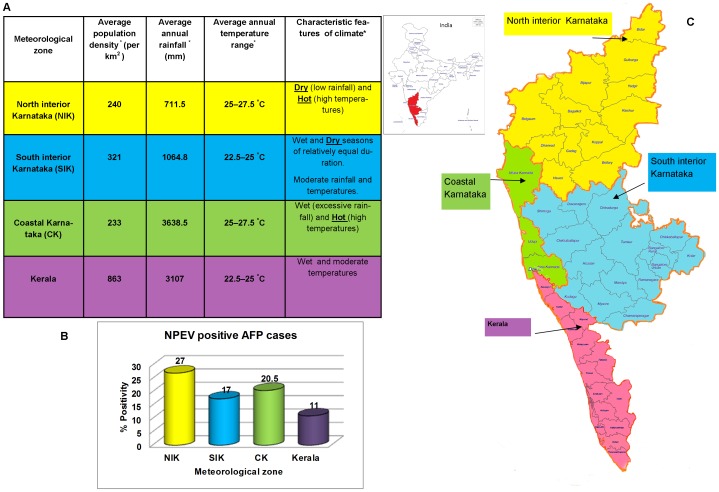
Meteorological zone wise analysis of NPEV positivity in AFP cases of the present study. **(A)** Demographic and climatological factors of different meteorological zones of south-western India. ^*^Retrieved from [25], [26], [27], [28], [39]. **(B)** Meteorological zone wise NPEV positivity. (**C**) Map of different meteorological zones of Karnataka and Kerala states of south-western India.

Based on meteorological parameters, Karnataka and Kerala states investigated in the study have been divided in four zones as north-interior Karnataka (NIK), south-interior Karnataka (SIK), coastal Karnataka (CK) and Kerala [25], [26], [27], [28]. NPEV positivity in AFP cases was found to be significantly higher in NIK (229/848; 27%) than in SIK (104/599; 17.4%, p<0.00002) and Kerala (72/656; 11%, p<0.0000001). However, it was not significantly different from CK (17/83; 20.5%, p>0.05). Both CK and SIK showed significantly higher NPEV positivity than Kerala (p<0.02/p<0.002) (Figure 2).

### VP1 sequence based genotyping of NPEV strains

**Figure 3:**
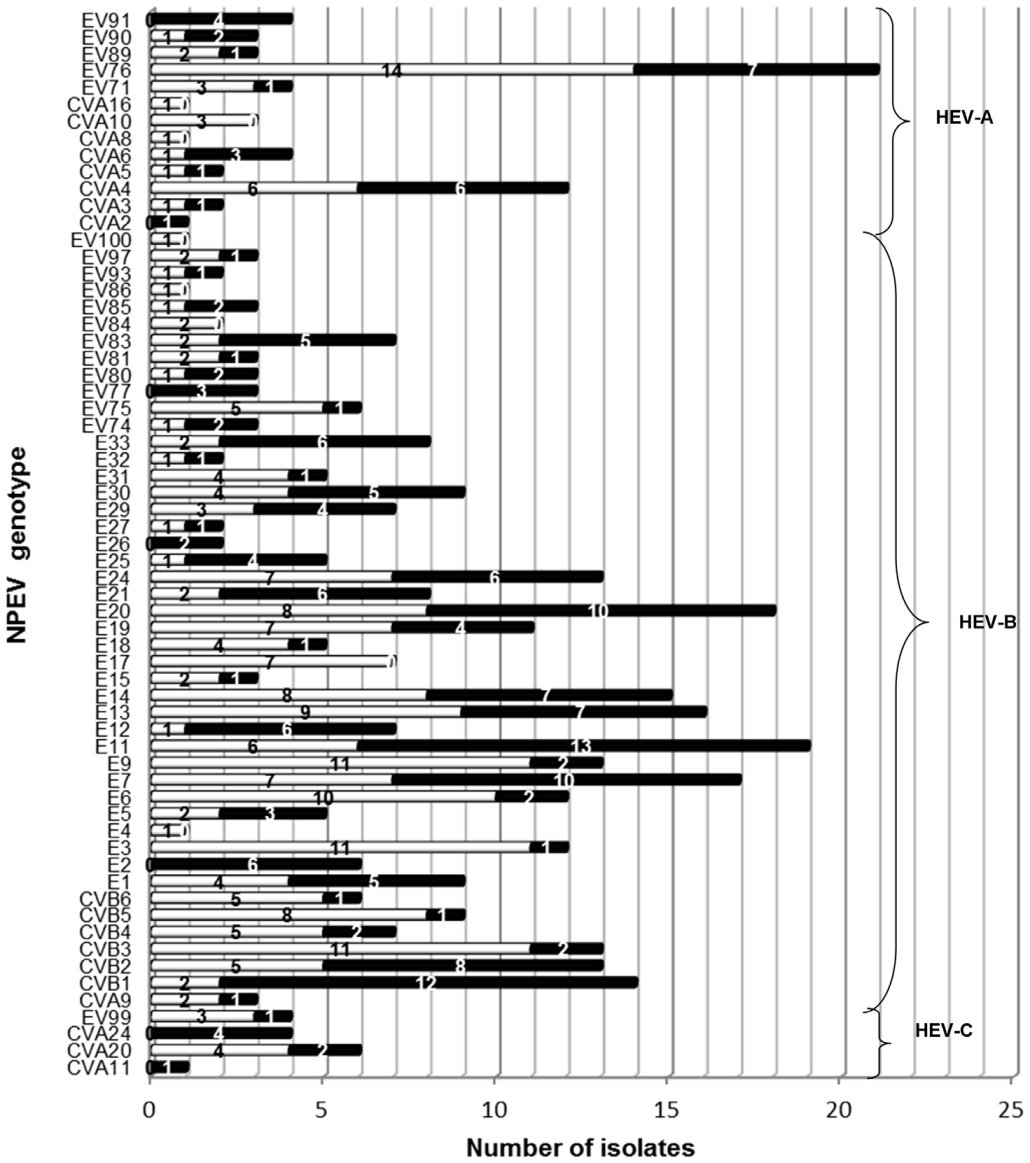
Distribution of NPEV types in AFP cases of the present study. □ and ▪ denote strains from 2009 and 2010 respectively.

Out of the 422 NPEV isolates from AFP cases, 415 were genotyped, while 7 remained untyped. Among genotyped isolates, 340 (81.9%), 60 (14.5%) and 15 (3.6%) showed presence of HEV-B (E: 238/340, 70%; CVB: 62/340, 18.2%; numbered EVs: 37/340, 10.9%; and CVA9: 3/340, 0.9%), HEV-A (CVA: 25/60, 41.7%; numbered EVs: 35/60, 58.3%) and HEV-C (CVA: 11/15, 73.3%; numbered EVs: 4/15, 26.7%) species respectively. Of the 63 different genotypes identified in AFP cases, EV76 (6.3%) and each of E3, CVB3 and E9 (4.97%) were found more frequently during 2009 while E11 (6.7%), CVB1 (6.1%), E7 (5.1%) and E20 (5.1%) were more common in circulation in 2010. CVA2, CVA11, CVA24, E26, EV77 and EV91 were not identified during 2009, but in 2010 (0.5–1.8%), while CVA8, CVA10, CVA16, E4, E17, EV84, EV86 and EV100 were identified during 2009 (0.5–3.6%), but not in 2010 (Figure 3).

Nineteen types of NPEV strains were found in association with 21 residual paralysis cases which included CVB6 and E24 (2 each), CVA4, CVA10, CVA11, CVA20, CVA24, CVB2, CVB3, CVB4, E7, E11, E12, E13, E14, EV75, EV76, EV83 and EV85 (1 each).

Nine NPEV types—CVB3 (5), EV80 (3), E30 and EV69 (2 each), CVB4, E6, E13, EV76 and EV81 (1 each) were isolated and identified from the 17 faecal specimens of the 41 asymptomatic contacts of AFP cases.

**Figure 4:**
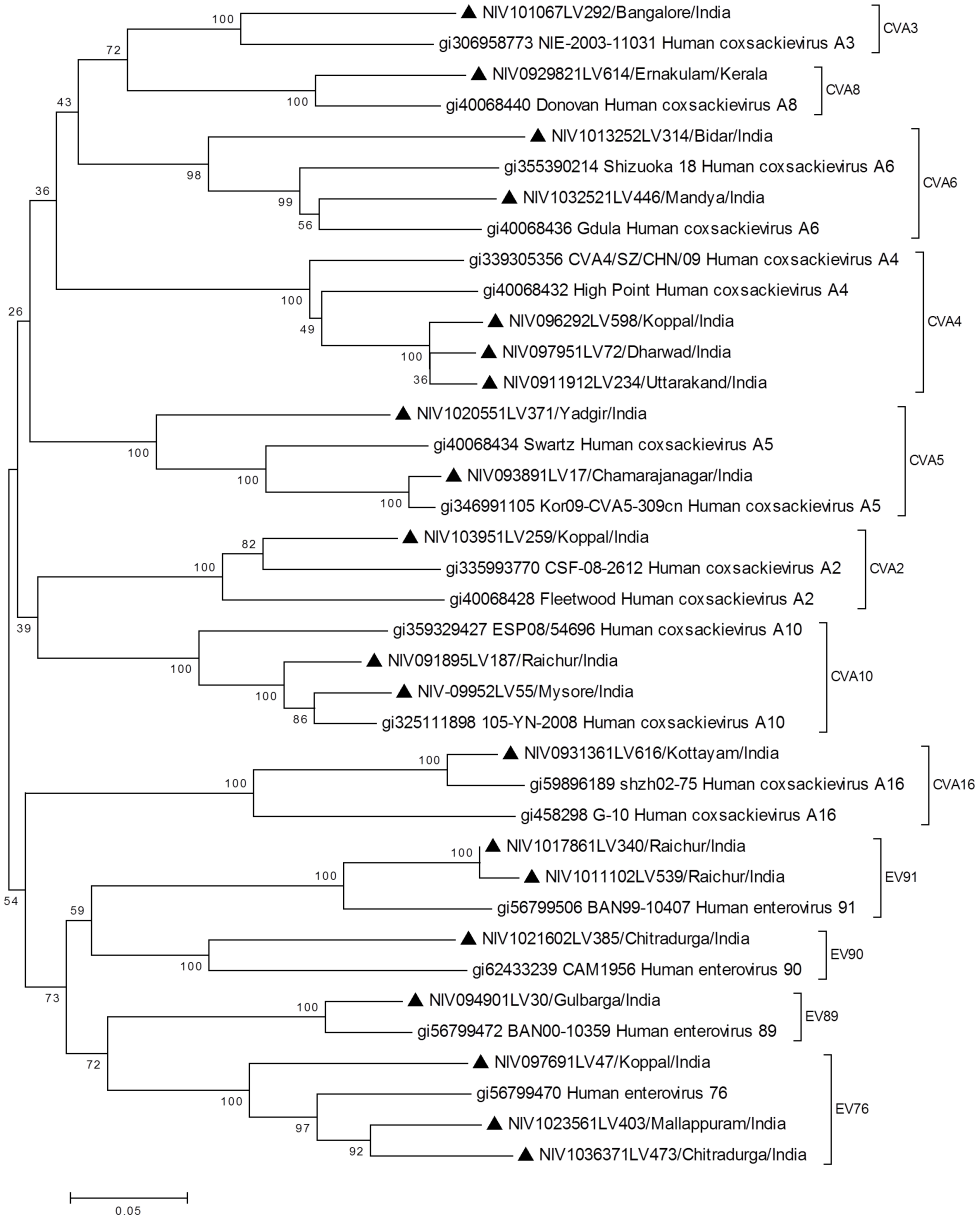
Phylogenetic tree based on VP1 sequence (nucleotide position: 2602–2977) of HEV-A strains of south- western India. ▴ denotes strain from AFP case of the present study. Reference strains are shown with GenBank accession numbers. Scale indicates genetic distance.

**Figure 5:**
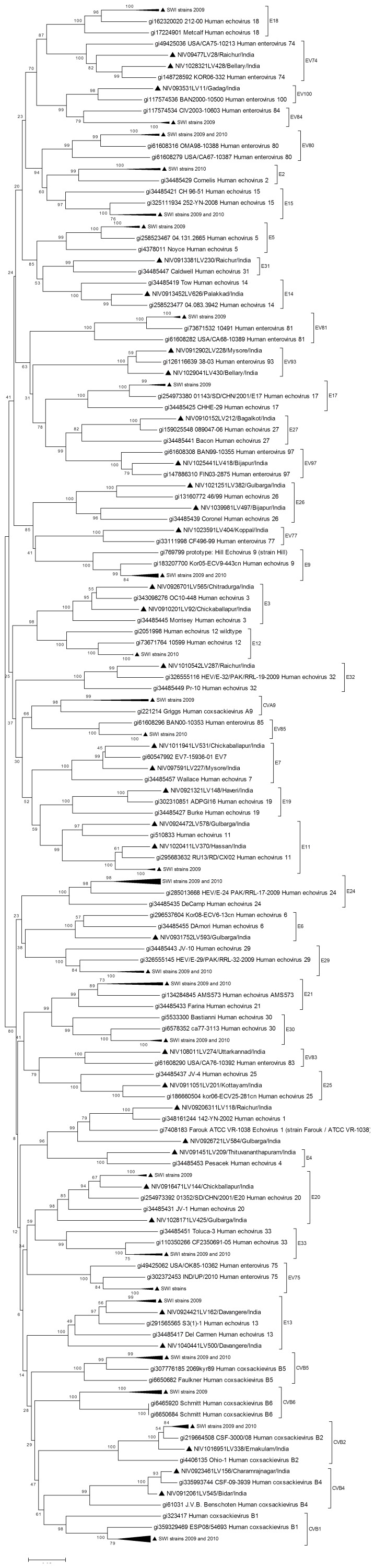
Phylogenetic tree based on VP1 sequence (nucleotide position: 2602?2977) of HEV-B strains of south-western India (SWI). ▴ denotes strain from AFP case of the present study. Strains which formed sub-clusters with >70% bootstrap support are collapsed. Reference strains are shown with GenBank accession numbers. Scale indicates genetic distance.

**Figure 6:**
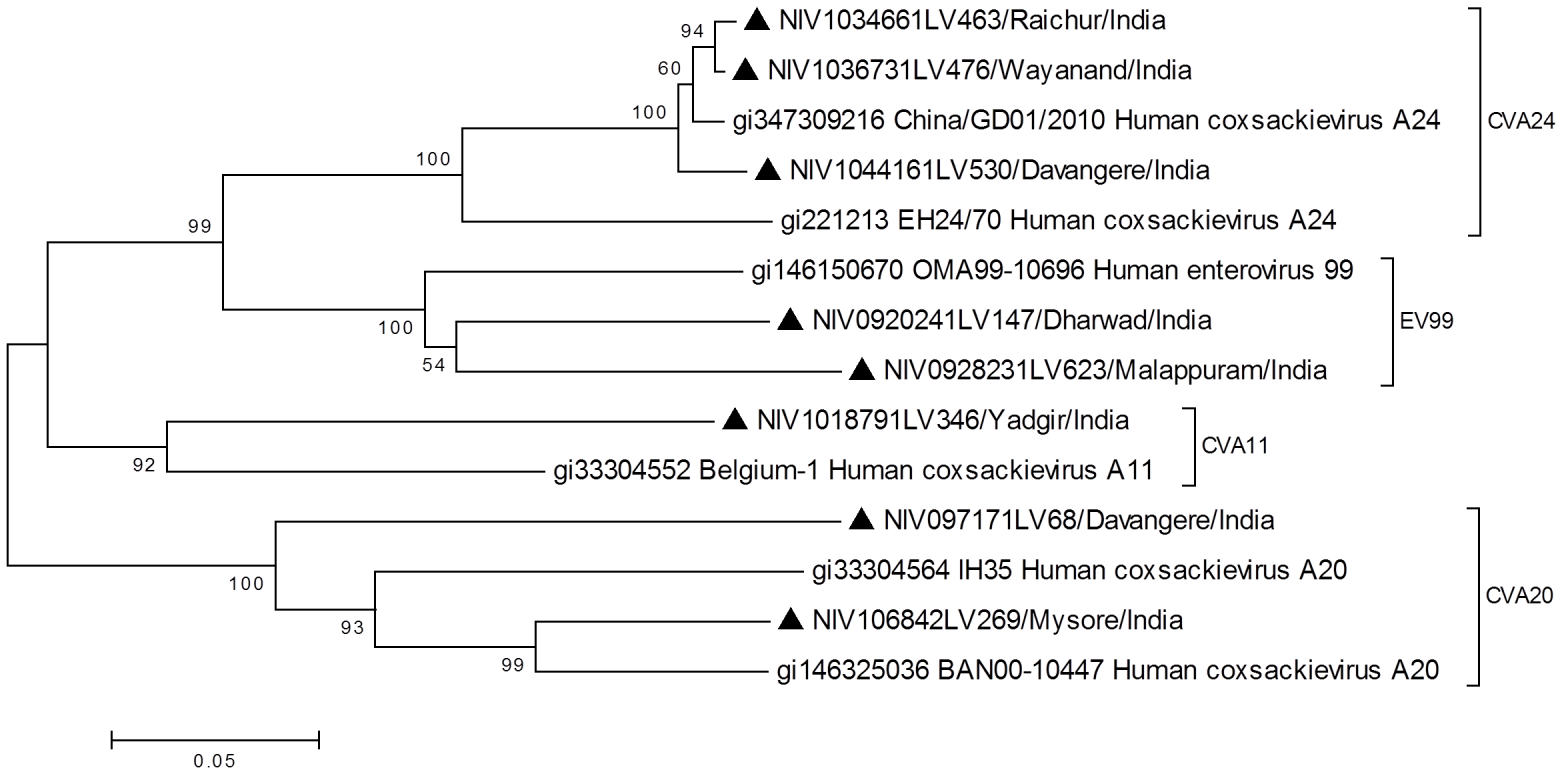
Phylogenetic tree based on VP1 sequence (nucleotide position: 2602–2977) of HEV-C strains of south- western India. ▴ denotes strain from AFP case of the present study. Reference strains are shown with GenBank accession numbers. Scale indicates genetic distance.

VP1 sequence (nucleotide position: 2602?2977) of all NPEV strains of this study showed highest identity (77?98%) with its counterpart in the NPEV strains circulating in close or distant geographical regions in the recent times and clustering with the respective prototype sequences in the phylogenetic analysis with bootstrap values indicated in the trees depicted in the Figure 4, Figure 5 and Figure 6. Almost all isolates formed monophyletic clusters with the exception of E1, E11, E14, E20 and EV76, which indicated co-circulation of strains of two different sub-clusters in the same year (data not shown).

Phylogenetic analysis of all 4 EV71 strains showed clustering with an Indian strain R13223-IND-01 (AY179600) (Figure S3), that has been classified in sub-genotype D [29] of the four sub-genotypes A, B, C and D described for EV71. All EV71 strains showed 90.6–93.8% identity with the reference strain (R13223-IND-01), and >15% divergence from the strains of other sub-genotypes. Nucleotide identity within the study strains was 91.4–94.9%.

VP1 gene sequence (nucleotide position: 2602–2977) of CVB3 strains isolated from one index AFP case and 5 asymptomatic contacts of the same case showed close (98.6–99.7%) genetic relatedness within themselves (Figure S4).

### Distribution of NPEV genotypes in AFP cases

CVA/CVB were found to be circulating significantly more in AFP cases aged ≥2–≤14 years than in <2 years (75/267; 28% vs 26/148; 17.6% p<0.02). E/numbered EVs prevailed more in AFP cases aged<2 years than in ≥2–≤14 years (122/148; 82.4% vs 192/267; 72%, p<0.02) (Figure S2A). On analysis of available clinical data, fever was found in a significantly low number of cases infected with CVA/CVB than with E/numbered EVs (31/87; 35.7% vs 142/274; 52% p<0.009) (Figure S2B). Circulation of CVA/CVB was found to be significantly more in AFP cases from Kerala (31/72; 43%) and CK (8/17; 47%) than those from NIK (39/222; 17.6%) (p<0.00002/p<0.009) and SIK (23/104; 22.1%) (p<0.004/p<0.05), whereas E/numbered EVs were more prevalent in AFP cases from NIK (183/222; 82.4%) and SIK (81/104; 77.9%) than those from CK (9/17; 53%) (p<0.009/p<0.05) and Kerala (41/72; 57%) (p<0.00002/p<0.004) (Figure S2C).

## Discussion

A striking rise in the non-polio AFP cases that has been reported during AFP surveillance carried out since 1997 in India for polio eradication [16], has vitalized the studies on the epidemiological and virological characteristics of NPEV infections associated with AFP cases in varied climatic zones.

In both years examined in the present study, NPEV positivity was detected through cell culture isolation. It was remarkably high (83.6% in 2009 and 80.6% in 2010) in children <5 years. This is in agreement with the studies from Pakistan and Romania reporting respectively 84% and 88% NPEV positivity among children aged between <1 and 5 years [30], [31]. Gender distribution (1.2 male:1 female) in NPEV positive AFP cases of the present study was almost similar to that described earlier from Pakistan (1.4:1) and Taiwan (1.7:1) [30], [32], however, differed from that of Austria and Tunisia reporting predominance in males [33], [34].

The available record of clinical data on AFP cases of the present study revealed occurrence of a variety of neurologic manifestations as sequel of NPEV infections (Figure S1). It is noteworthy that a significant proportion (38.6%) of the NPEV positive cases (n = 88) was classified to have GBS. Similar data has been documented earlier for NPEV associated AFP cases from Pakistan and Americas [30], [35]. Twenty per cent of the NPEV positive cases of the present study were diagnosed to have traumatic neuritis (Figure S1). Coincidental occurrence of this non-infectious clinical manifestation [4], [36], though cannot be explained due to limited information on these cases, has been also noted earlier in AFP cases caused by PV [37].

Fever at the onset of paralysis is one of the cardinal signs of poliomyelitis [35]. It was also found to be a frequent symptom in NPEV infection in the studies from northern India, Pakistan, Taiwan and Brazil [13], [30], [32], [38]. However, in the present study, this symptom has not shown significant association with NPEV infection. This finding is parallel to that obtained for the NPEV associated AFP cases reported from Americas [35].

The activity of NPEVs recorded in this study was found to be high during summer months. This observation was closer to that described in earlier studies from northern India and Americas [14], [35]. It has been reported that the non-polio AFP rate was not associated with population density [10]. In concurrence with this finding, NPEV positivity in AFP cases of the present study was noted to be remarkably high in NIK and CK known to have lower population densities [39], as compared to that of densely populated SIK and Kerala (Figure 2). Further, a significantly lower NPEV positivity in AFP cases was noted in the relatively cooler climate (Figure 2). The climatic factors may thus affect the transmission and infection rates of NPEVs as has been assumed earlier [14], [33]. AFP cases with residual paralysis were recorded in all of the meteorological zones during follow-up examination. Interestingly, a marked association (55.6%) of NPEV positivity with residual paralysis was noted in Karnataka but not in Kerala state. This could be incidental finding, however, it suggests that the determinants of virulence of NPEVs and also the host immunity in NPEV associated AFP cases, identified under different climatic conditions need to be studied.

Analysis of VP1 gene sequences of NPEV strains is widely carried out to determine the HEV species and genotypes that circulate in the population [21], [40]. According to this strategy, predominance of HEV-B species found in this study was similar to that of the recent reports described for NPEV strains from hospitalized children and AFP cases from Nigeria and Philippines [41], [42]. A significantly higher proportion of AFP cases with E/numbered EV infection than that identified with CVA/CVB infection was found in children aged <2 years; with fever; and in NIK and SIK regions (Figure S2). Interestingly, fever at the onset of paralysis showed no significant relationship with any age group (data not shown), although its occurrence was found in association with E/numbered EV infection. Overall, these data highlight the importance of analysis of epidemiological and clinical characteristics of NPEV infections in combination with molecular typing of NPEVs.

Identification of a multitude of NPEV isolates in the present study (Figure 3) has supported the susceptibility of the RD cell line to almost all types of the NPEVs including CVB, CVA9, CVA24, E9 and E11 described to be isolated at a low rate [43]. However, this might be a partial representation of circulating NPEVs, as none of the other cell systems was utilized in the study. Nevertheless, the data obtained in this study affirm the association of diverse NPEV genotypes with AFP as reported by other investigators [15], [41], [42]. E11, EV76, E20, E7, E3, E9, CVB1 and CVB3 were identified frequently in this study. Of these, E11 has been also found to be prevalent in AFP cases from Romania, northern India and Finland [31], [44], [45]. Based on the partial VP1 gene analysis, all NPEV strains in this study demonstrated considerable diversity within each type (0–20%), with respective prototype (10–25%) and recent reference (2–23%) strains. The extreme plasticity known for the single stranded RNA genome of NPEVs could explain this observation.

EV71, known for its etiological role in causing epidemics of severe neurological diseases [46] was detected in four cases of AFP from interior parts of Karnataka. All four strains differed phylogenetically (Figure S3) from the sub-genotypes detected frequently in Hand Foot and Mouth Disease [47], [48]. It is to be noted that the genotypes identified in the residual paralysis cases were also found in the recovered cases. NPEV strains were also isolated from the asymptomatic contacts investigated in the study. All types detected in asymptomatic contacts, except EV69 (described to be not associated with any disease [49]), were found in association with AFP. CVB3 strains detected in an AFP case and corresponding 5 asymptomatic contacts differed within themselves by 0.3% to 1.4% in the VP1 region analyzed in the study (Figure S4). Full length genome sequence analysis of these strains would help to identify variations in other genomic regions, if any, associated with the severity of disease.

While most of the EV infections are asymptomatic, a few of the infections may lead to AFP [5]. Although detection of one of the NPEV types during the course of AFP may not provide a proof of a causal relationship, the etiological role of NPEV associated with this syndrome also may not be ruled out.

To summarize, the present study underscores the possible link between the circulation pattern of NPEVs and AFP in different populations and climatic conditions in south-western India during 2009–2010. The genetic analysis of NPEV strains carried out in the study has confirmed the extensive diversity in NPEVs circulating in the AFP cases. No single NPEV type was dominant consistently, although a large number of genotypes was detected. This study suggests the need to investigate PV negative NPEV positive AFP cases from different meteorological zones of India for better understanding of NPEV infections and contribution of viral and host factors influencing these infections, in the post-polio eradication era. The study also endorses the confidence in the Poliomyelitis Eradication Initiative, its clinical and laboratory investigations, along with the algorithm for testing and reporting.

## Supporting Information

Figure S1 **Distribution of clinical manifestations of NPEV positive AFP cases of the present study.**
(TIF)

Figure S2 **Circulation of NPEV genotypes in AFP cases from different age groups, clinical features and meteorological zones.**
(TIF)

Figure S3 **Phylogenetic tree based on VP1 sequence (nucleotide position: 2602–2977) of EV71 strains of south-western India.**
(TIF)

Figure S4 **Phylogenetic tree based on VP1 sequence (nucleotide position: 2602–2977) of CVB3 strains of south-western India.**
(TIF)
