# Supplementary figures and images for: Characterization of the Non-Polio Enterovirus Infections Associated with Acute Flaccid Paralysis in South-Western India
Source: PLoS One. 2013 Apr 22;8(4):e61650. doi: 10.1371/journal.pone.0061650 (PMC3632520; doi:10.1371/journal.pone.0061650)

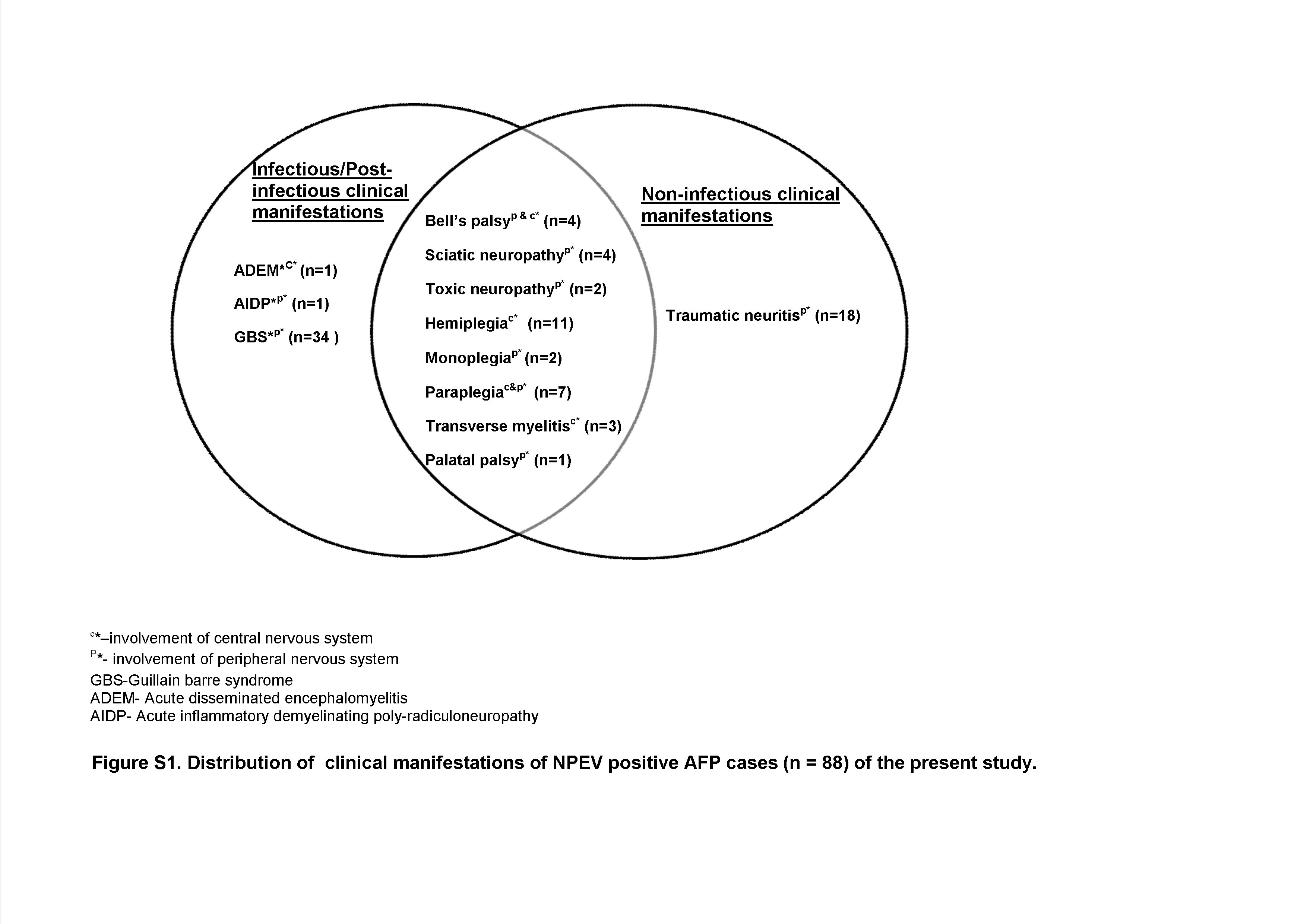

Supplement: Figure S1 — Distribution of clinical manifestations of NPEV positive AFP cases of the present study. (TIF) [file pone.0061650.s001.tif]

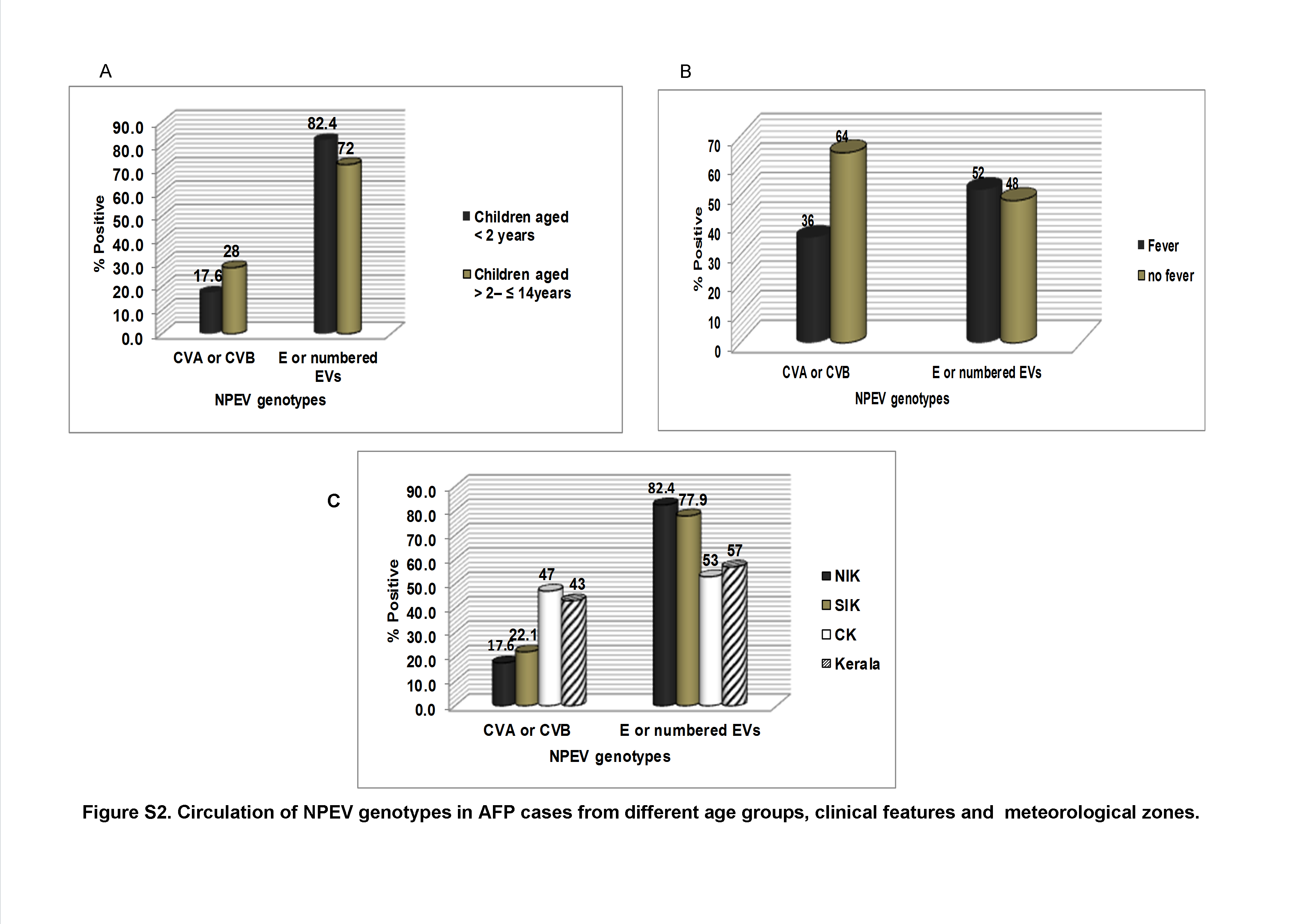

Supplement: Figure S2 — Circulation of NPEV genotypes in AFP cases from different age groups, clinical features and meteorological zones. (TIF) [file pone.0061650.s002.tif]

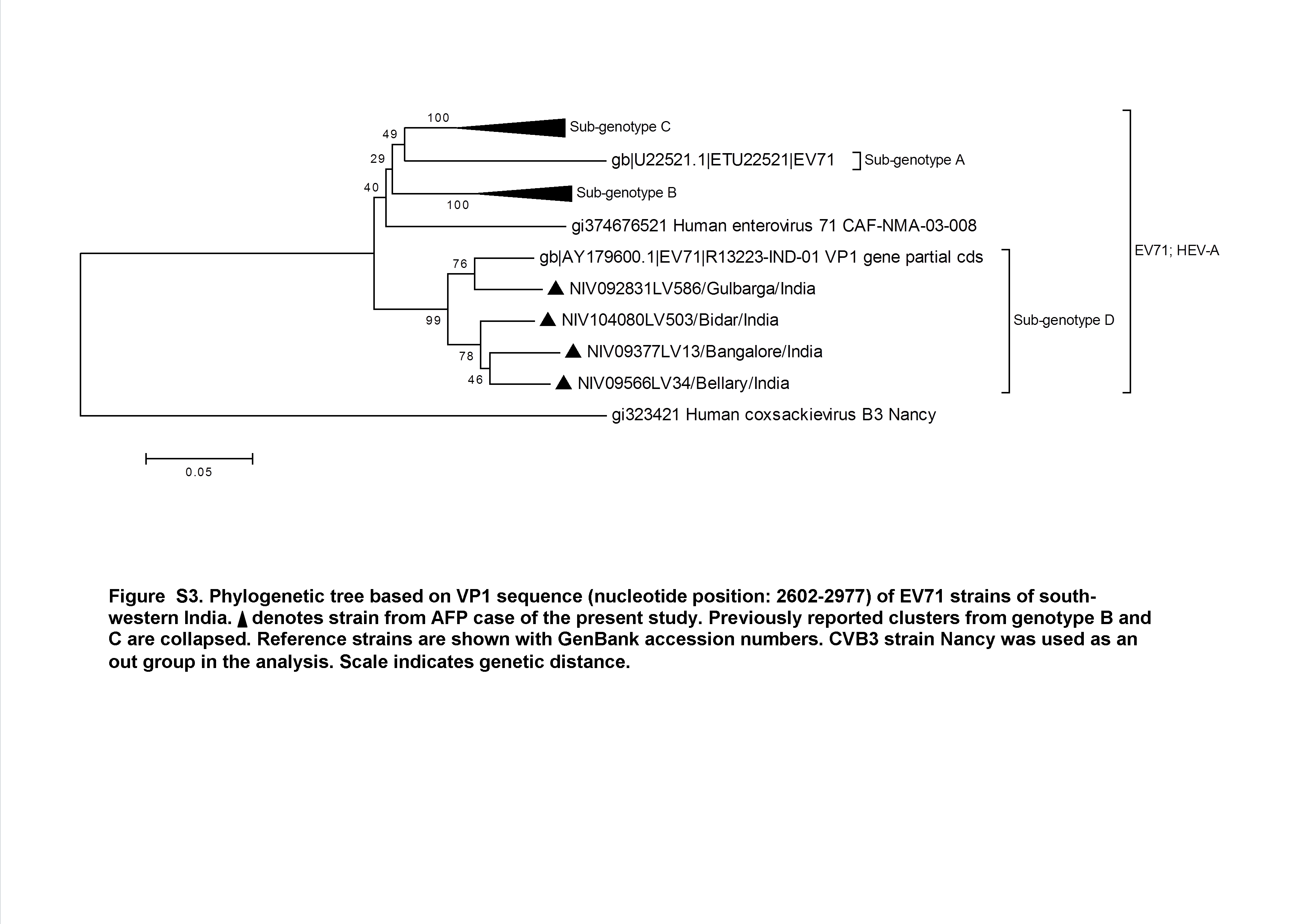

Supplement: Figure S3 — Phylogenetic tree based on VP1 sequence (nucleotide position: 2602–2977) of EV71 strains of south-western India. (TIF) [file pone.0061650.s003.tif]

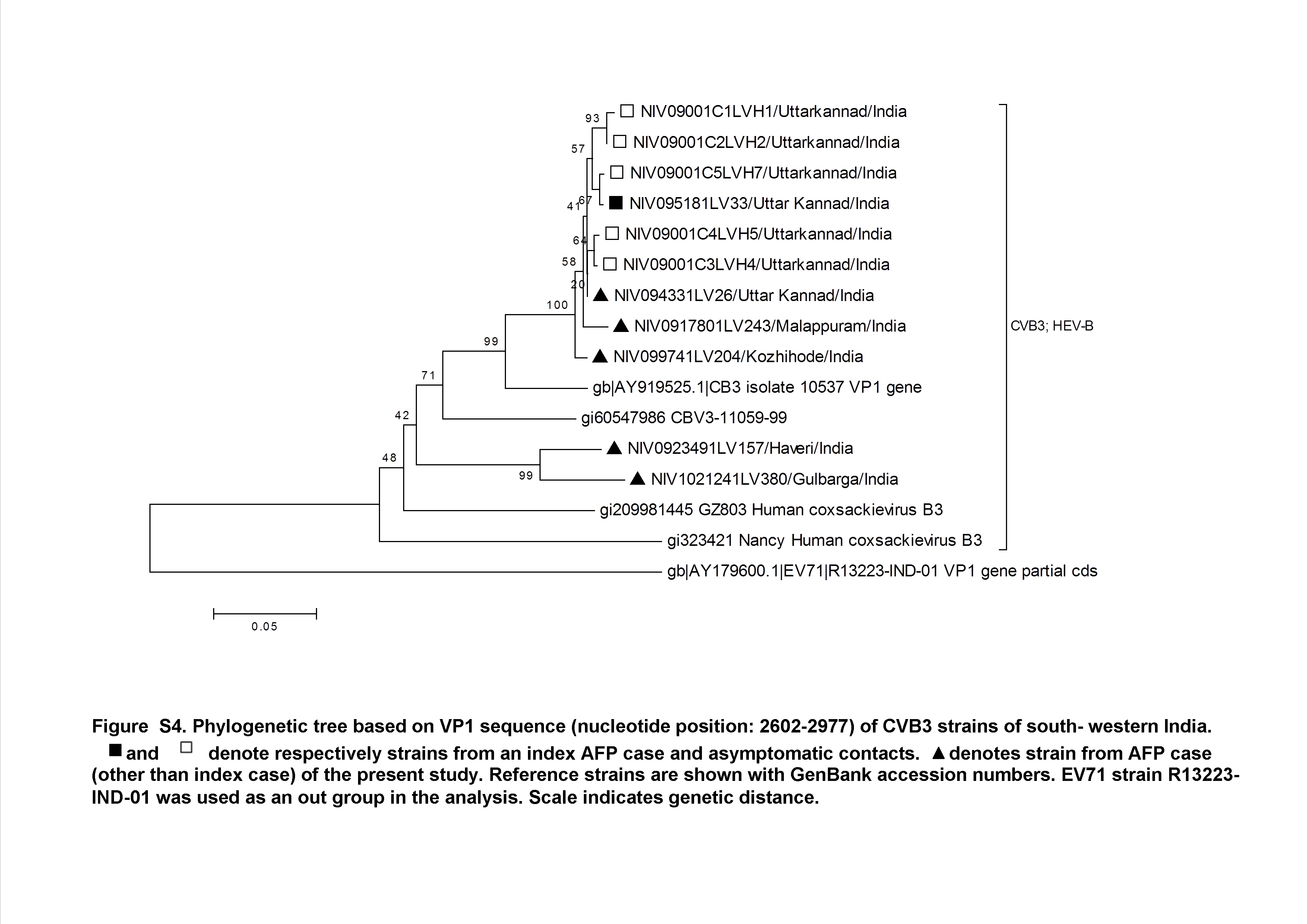

Supplement: Figure S4 — Phylogenetic tree based on VP1 sequence (nucleotide position: 2602–2977) of CVB3 strains of south-western India. (TIF) [file pone.0061650.s004.tif]
